# Coarse-Grained Approach to Simulate Signatures of Excitation Energy Transfer in Two-Dimensional Electronic Spectroscopy of Large Molecular Systems

**DOI:** 10.1021/acs.jctc.4c00413

**Published:** 2024-07-12

**Authors:** Kai Zhong, Hoang Long Nguyen, Thanh Nhut Do, Howe-Siang Tan, Jasper Knoester, Thomas L. C. Jansen

**Affiliations:** † Zernike Institute for Advanced Materials, University of Groningen, Nijenborgh 3, 9747 AG Groningen, The Netherlands; ‡ School of Chemistry, Chemical Engineering and Biotechnology, Nanyang Technological University, 21 Nanyang Link, 637371 Singapore; ¶ Faculty of Science, Leiden University, Einsteinweg 55, 2300 RA Leiden, The Netherlands

## Abstract

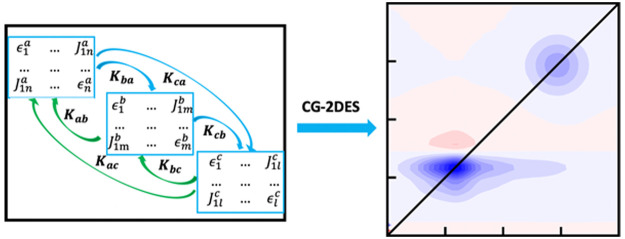

Two-dimensional electronic spectroscopy (2DES) has proven to be a highly effective technique in studying the properties of excited states and the process of excitation energy transfer in complex molecular assemblies, particularly in biological light-harvesting systems. However, the accurate simulation of 2DES for large systems still poses a challenge because of the heavy computational demands it entails. In an effort to overcome this limitation, we devised a coarse-grained 2DES method. This method encompasses the treatment of the entire system by dividing it into distinct weakly coupled segments, which are assumed to communicate predominantly through incoherent exciton transfer. We first demonstrate the efficiency of this method through simulation on a model dimer system, which demonstrates a marked improvement in calculation efficiency, with results that exhibit good concordance with reference spectra calculated with less approximate methods. Additionally, the application of this method to the light-harvesting antenna 2 (LH2) complex of purple bacteria showcases its advantages, accuracy, and limitations. Furthermore, simulating the anisotropy decay in LH2 induced by energy transfer and its comparison with experiments confirm that the method is capable of accurately describing dynamical processes in a biologically relevant system. This method presented lends itself to an extension that accounts for the effect of intrasegment relaxation processes on the 2DES spectra, which for computational efficiency are ignored in the implementation reported here. It is envisioned that the method will be employed in the future to accurately and efficiently calculate 2D spectra of more extensive systems, such as photosynthetic supercomplexes.

## 1 Introduction

Two-dimensional electronic spectroscopy^1^ (2DES) elucidates the dynamics in advanced materials and biological systems at the femtosecond time scale and currently plays a vital role in the research on energy transport in such systems.^2−15^ In these complex systems, it is challenging to unravel the intricate details of the dynamic processes, which is important to understand and possibly improve their functionality. The ability to simulate 2D spectra is essential to gain a better understanding as well as to achieve improved functionality of large molecular systems. However, for such large complex systems, this remains a formidable task due to the unfavorable computational scaling with system size.^16^ Therefore, efficient calculation schemes are needed. This paper aims to develop an efficient simulation protocol for 2DES that provides accurate results at a small computational cost, even for an extensive system. This is achieved by coarse-graining the system into manageable segments.

Considerable efforts have been devoted to enable the simulation of two-dimensional spectra for large molecular systems. For instance, the generalized quantum master equation (GQME) method and its extended variant rely on correlation functions to extract the multidimensional spectroscopic signals.^17−19^ Another widely recognized technique for accurate calculations of spectra is the Hierarchy Equations of Motion (HEOM) method, frequently employed as a benchmark.^20−23^ However, the obvious drawback is that the HEOM method is too computationally expensive, particularly for large systems due to a factorial scaling with system size. Efforts have, however, been made to make the method computationally more tractable by the use of graphical processing units (GPU).^24^ For the related Hierarchy of Pure States (HOPS)^25^ based on wave functions, which suffer from the same type of scaling issues as HEOM, the use of an adaptive hierarchy^26^ allows for a significant speedup and improved scaling. One further method to simulate 2D spectra is the Numerical Integration of the Schrödinger Equation (NISE) approach.^27^ While the computational time for this method scales more favorably with system size, its original version required diagonalizing the two-particle Hamiltonian.^3^ The implementation of more efficient propagation schemes in NISE, taking into account the typical structure of the two-exciton part of the Hamiltonian^28,29^ and its sparse nature,^30^ eventually reduced the overall scaling of the method to an *N*^3^ dependence on the number of chromophores, *N*. Further implementing^31^ parallel computing algorithms combining a message passing interface (MPI) and open multiprocessing (OpenMP), the application of NISE to large systems with thousands^32,33^ of coupled chromophores was realized. However, such calculation with the use of hundreds or even thousands of computer cores still takes multiple days. It is, therefore, still crucial to develop more efficient approaches for calculating 2D spectra for large systems relevant to biological and materials sciences.

Here, we develop a Coarse-Grained 2DES (CG-2DES) computational method, which is computationally efficient, especially for large systems. This method divides the full system into small segments based on the intermolecular coupling strengths and calculates contributions to the 2DES separately for each segment.^34,35^ The individual segments are interconnected using a rate equation to describe the exciton transfer between them. We calculate the required intersegment rate matrix using the Time-Domain Multichromophoric Förster Resonance Energy Transfer (TD-MCFRET) method.^36^ In the CG-2DES method, we assume the excitation within one-segment to equilibrate much faster over its constituent molecules than the time scale of the population transfer between different segments. Furthermore, we include the possibility of accounting for a detailed balance in the thermal relaxation process, which allows for the accounting of thermal relaxation in the 2DES.

This paper is organized as follows. In the following section, we will first discuss the theory to model the system and the algorithm for calculating 2D spectra. The implementation of the CG-2DES method is described in Section 3. In Section 3.1 and in Section 3.2, spectral results for different model systems are demonstrated and analyzed. The application to the LH2 system is presented in Section 3.3. Finally, in the last section, we will draw our conclusions.

## 2 Theory and Method

### 2.1 Model System

First, we introduce our Hamiltonian. In our approach, we assume that all molecules in the assembly are well described as two-level systems with a ground state and an optically accessible excited state. As long as the charge densities of the excited states of different molecules do not overlap, the delocalized collective excited states of the assembly can be described as Frenkel excitons.^37^ Our goal is to simulate the 2DES, allowing for a large number of molecules (10s to 1000s) and accounting in an efficient way for exciton dynamics, in particular for excitation energy transport through the entire system. A full calculation, treating all intermolecular interactions on equal footing, is computationally prohibitively expensive for large systems. In our method, we take advantage of the fact that in many large molecular systems, segments may be distinguished such that the interactions between segments are relatively weak, much weaker than those within segments. As a result, the excitation energy transfer between segments may be considered as incoherent jumps on a time scale that is large enough to consider the excitation within individual segments as completely equilibrated. This allows for a great gain in computational cost. Namely, the 2DES of the entire system may then be obtained by calculating the spectra of separate segments and adding those with appropriate weights. The latter follows from solving a set of rate equations, dictated by energy transfer rates, which we will calculate using the TD-MCFRET method,^36^ a trajectory-based version of the MCFRET method.^38^ We segment the full system into different parts such that intersegment intermolecular interactions are small and excitation energy transfer (EET) can be treated as incoherent. We will define the Hamiltonian for one segment, and the sum of these one-segment Hamiltonians and the Hamiltonian for the interactions between segments gives the full Hamiltonian.

**Figure 1:**
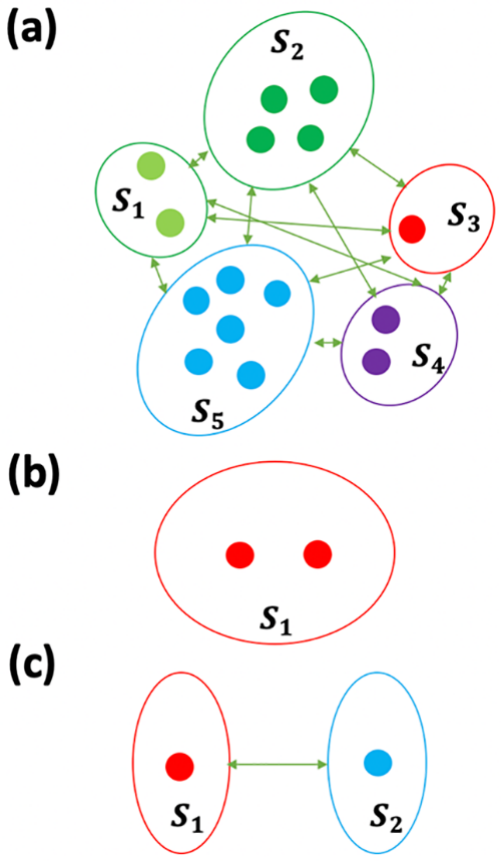
Illustration of the segmentation scheme. (a) Example of segmentation of a large system into five individual segments labeled with different colors. (b) A dimer within one segment. (c) A dimer system is divided into two individual segments.

Thus, we start by defining the Frenkel exciton Hamiltonian for a single segment, labeled *S*_*i*_ (See Figure 1 (a)):
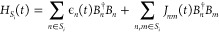
1Here, *n* and *m* represent different molecules belonging to segment *S*_*i*_, ϵ_*n*_(*t*) is the excitation energy of molecule *n*, and *J*_*nm*_(*t*) is the coupling between molecules *n* and *m. B*_*n*_^†^ and *B*_*n*_ are Pauli creation and annihilation operators.^39^ The time-dependence of the site energies and interactions are included to describe the effects of fluctuations in these parameters due to the coupling to a dynamic environment, modeled as a classical bath (see below). The interaction between two-segments, *S*_*i*_ and *S*_*j*_, can be expressed as
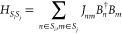
2Here, we assume that these interactions may be considered time-independent. This facilitates the use of our TD-MCFRET method. Within the segments, we will consider fluctuations because these are important in describing spectral broadening effects in an appropriate way. As our method to deal with fluctuations is phenomenological, in our present simulations we will restrict ourselves to fluctuations in the site energies only. In principle, generalization of this method to time-dependent interactions is possible,^36^ but such a generalization would make the calculations more time-consuming. Furthermore, it was found that the coupling fluctuations are typically an order of magnitude smaller than the frequency fluctuations.^40^ With this justification, we assume that these interactions are already small; normally, fluctuations will only be a negligible correction to an already small number. The systems of our interest typically have a fairly fixed structure, and the couplings are determined by the mutual spatial arrangement of the chromophores. This may, however, be a more critical issue when including charge transfer states, where couplings have a more sensitive distance dependence or when transition dipoles with near perpendicular dipoles are present.^41^ Thus, in eq 1 we will replace *J*_*nm*_(*t*) by *J*_*nm*_, time-independent model parameters which we will specify in the various applications of our method considered in Section 3.

The distinction between a collection of quantum degrees of freedom and the environment is rooted in the system-bath separation, a well-used model for such a complex system. In this paper, we prefer to use a simple system-bath interaction to test our method. Therefore, overdamped Brownian oscillators, which have a limited number of free parameters, are used to describe this interaction.^42^ The excitation energy has a linear dependence on the bath degree of freedom, effectively reducing the bath description to be governed by a magnitude of the energy fluctuations, σ, and a correlation time, Λ^–1^, describing the memory loss of these fluctuations. Here, we neglect the influence of electronic excitation on the bath dynamics. Therefore, for example, the Stokes shift is neglected in the emission. It could be added in an approximate way similar to how it was done in other approaches^43^ at an additional computational cost. We note that the method developed here allows for far more complex system-bath couplings going beyond the spectral density approximation as, for example, obtained using molecular dynamics (MD) simulations to describe the bath dynamics^27^ in combination with *ab initio*(27) and mapping methods.^44^ One could alternatively use the cumulant expansion^45^ to obtain lineshapes, which was first applied to the doorway-window picture to describe the interaction between the system and the bath. Our method is thus not limited to the treatment of an uncorrelated harmonic bath.

The bath dynamics is, thus, fully characterized by the time-correlation function:
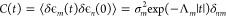
3where σ_*m*_ denotes the root-mean-squared magnitude of the energy fluctuations for molecule *m*, which we assume to be uncorrelated for different molecules, and Λ_*m*_ is the inverse of the correlation time. More details for the bath fluctuations can be found in ref (46).

### 2.2 Two-Dimensional Electronic Spectroscopy

**Figure 2:**
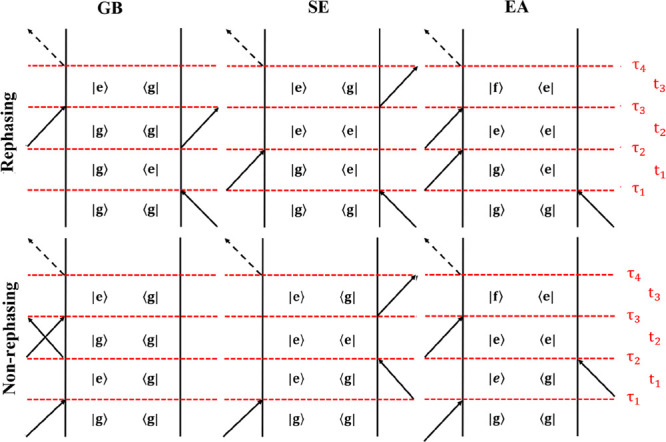
Double-sided Feynman diagrams contribute to the 2DES signal. The top figures illustrate the rephasing components of the GB, SE, and EA, while the bottom ones show the corresponding nonrephasing signals. Here, |g⟩, |e⟩, and |f⟩ denote the quantum ground state (no excitons) and excitations in the singly and doubly excited manifold, respectively. The full arrows represent the interactions with the applied light pulses, and the dashed arrows represent the generated signals. On the right side, the times τ_1_, τ_2_, τ_3_, and τ_4_ connected with the red dashed lines specify the interaction times, and the relevant intervals between these interactions *t*_1_, *t*_2_, and *t*_3_, are illustrated as well.

2D spectra involves the interaction of three light pulses with the exciton system, resulting in the generation of a signal electric field. The total signal is the sum of signals emitted in various directions described by their wave vectors. We shall focus on the signals emitted in the directions with wave vectors *k⃗*_*I*_ = −*k⃗*_1_ + *k⃗*_2_ + *k⃗*_3_ and *k⃗*_*II*_ = *k⃗*_1_ – *k⃗*_2_ + *k⃗*_3_, where *k⃗*_1_, *k⃗*_2_, and *k⃗*_3_ are the wave vectors of the incoming light pulses. *k⃗*_*I*_ and *k⃗*_*II*_ are also referred to as rephasing and nonrephasing pathways, respectively. We may demarcate the signals into three contributions: the ground-state bleach (GB), stimulated emission (SE), and excited-state absorption (EA) signals, each of which has a rephasing and a nonrephasing component.^47^ The double-sided Feynman diagrams associated with these contributions are given in Figure 2.

As depicted in Figure 2, the pulse interactions are associated with the times *t* = τ_1_, τ_2_, τ_3_, and τ_4_, while the intervals between the successive interactions are denoted *t*_1_, *t*_2_, and *t*_3_. The times *t*_1_ and *t*_3_ are denoted coherence times, as the system is in coherence between the ground state and a singly excited state or between a doubly excited state and a singly excited state during *t*_3_ for the EA diagrams. The time *t*_2_ is known as the population time or waiting time; varying *t*_2_ allows studying the dynamics in the ground state and singly excited state. The response functions for the GB, SE, and EA signals are given by^48^
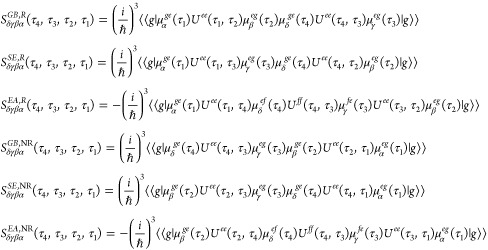
4

The brackets ⟨⟨ ··· ⟩⟩ represent the average over the classical ensemble and the expectation value of the quantum system together. The time evolution operator, *U*^*gg*^(τ_*x*_, τ_*y*_), for the ground states is omitted since it is just the identity operator. Here, we represent the transition dipole moment between the initial ground state (denoted |*g*⟩) and a state in the single excited manifold (denoted |*e*⟩) as μ_*X*_^*ge*^, the transition dipole moment between the first excited manifold and the second excited manifold (denoted |*f*⟩) is labeled with μ_*X*_^*ef*^. The subscript X can take the values α, β, γ, δ, which specify the polarization of the relevant electric field. The superscript in the propagator (*ee*)/(*ff*) denotes the propagation when there is a single/double excitation in the quantum system.

The signals are converted to the frequency domain (ω_1_ and ω_3_) using double Fourier transforms with respect to the coherence times *t*_1_ and *t*_3_, respectively, for a given “waiting time” *t*_2_. In practice, to obtain the 2DES, the response can be calculated independently on the basis of the single excitation manifold and the double excitation manifold, as those do not couple when no external electric field is present. There are *N* single excited states and *N*(*N* + 1)/2 double excited states. With the propagation scheme used in the NISE method,^30^ the cost for calculating the time-evolution operator for the single excitation manifold is proportional to the *N*^3^. The calculation cost for the double excitation manifold is proportional to *N*^6^. By applying the Trotter approximation and using sparse matrix techniques,^30^ we can reduce the calculation cost for the propagation of the double excitation manifold from *N*^6^ to *N*^3^.

**Figure 3:**
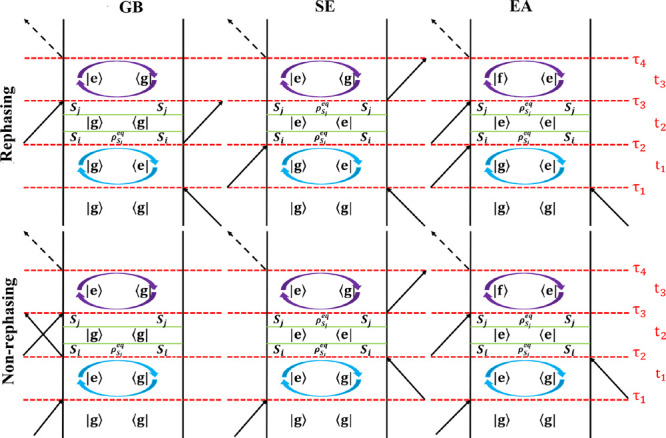
CG-2DES double-sided Feynman diagrams contributing to the 2DES signal. The symbols for the interaction times, time delays, arrows, and states are the same as in Figure 2. ρ_*S*_*i*__^eq^ (blue elliptical arrows) and ρ_*S*_*j*__^eq^ (purple elliptical arrows) represent the equilibrium within the segments *S*_*i*_ and *S*_*j*_, respectively. It is assumed that segment *S*_*i*_ equilibrates to the state described by ρ_*S*_*i*__^eq^ before the transfer can happen to segment *S*_*j*_. The exciton transfer from the segment *S*_*i*_ to *S*_*j*_ happens during the waiting time *t*_2_, which is illustrated as the period between the two green lines. It is assumed that the population on segment *S*_*j*_ equilibrates to the state described by ρ_*S*_*j*__^eq^ in the time interval after the transfer to this segment has happened but before the detection initiated by the interaction at τ_3_.

In the NISE approach, one needs to propagate the full system around the full diagrams, which is time-consuming for large systems and for long waiting times. For the coarse-grained two-dimensional electronic spectroscopy (CG-2DES), we calculate the response function in a different way. The Feynman diagrams for the six contributions distinguished in Figure 2 are illustrated again in Figure 3, using the concept of segmentation. We will explain the principle in detail for the nonrephasing diagrams, as an example, and only provide the final equations for the remaining diagrams. Following eq 4, the string of operators is rewritten from back to front (taking the complex conjugate), and we split all the time evolution operators into individual ones. Furthermore, we trace over the space of all (multi)exciton states. This results in the ⟨*g*| ··· |*g*⟩ being replaced with a Tr. The nonrephasing EA signal may thus rewritten as
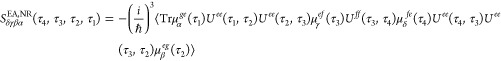
5where the remaining angular brackets ⟨ ··· ⟩ denote the average over the bath degrees of freedom. Next, by cyclic reordering of eq 5, which is allowed in the trace, we obtain
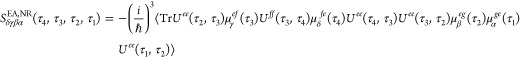
6

To include exciton population relaxation, we recast the response function in the doorway-window picture formalism.^45^ Here, we assume that the transfer between segments only happens during the waiting time, which is justified if couplings between segments are small and the transfer is slow compared to the typically short coherence times. We can then separate the equation into three parts, one for each time interval. The signal contribution during *t*_1_ is denoted the doorway part. Isolating the dynamics during *t*_1_ which only involves segment *S*_*i*_, we have the doorway function:
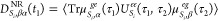
7Here, μ_*S*_*i*_,α_^*ge*^ are transition dipoles for exciton states on segment *S*_*i*_ and *U*_*S*_*i*__^*ee*^(τ_1_, τ_2_) is the time-evolution matrix corresponding to the Hamiltonian *H*_*S*_*i*__(*t*) for segment *S*_*i*_. By examining all nonrephasing diagrams, we see that the time evolution is the same for all diagrams.

Next, we move to the waiting time, *t*_2_. Here, we assume that the system will first reach the equilibrium in segment *S*_*i*_. Energy transfer to another segment *S*_*j*_ may then occur. After which, the system will re-equilibrate on segment *S*_*j*_ before detection (see Figure 3). The result of the exciton transfer process is represented by the probability of the exciton being found on segment *S*_*j*_ at a time interval *t*_2_ after it started on segment *S*_*i*_. This is given by the matrix elements of the transfer function
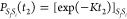
8Here, *K* is the rate matrix for transfer between all involved segments, which we will obtain from the TD-MCFRET method.^36^ In principle, one may also apply our method by using a rate matrix obtained in another way or by using a phenomenological rate matrix. To ensure that the rate matrix fulfills the detailed balance, we apply the standard thermal correction factor^49^ on the elements of the rate matrix
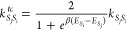
9with β=, where *k*_B_ is the Boltzmann constant and *T* the temperature. *E*_*S*_*j*__ and *E*_*S*_*i*__ represent the expectation value of the energy in thermal equilibrium in segment *E*_*S*_*j*__ and *E*_*S*_*i*__, respectively. The expectation value for the energy of segment *S*_*j*_ is determined by the ensemble average over the full trajectory^50^
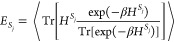
10

To avoid overcorrection, the segment energies used in the thermal correction can be adjusted by *ΔE*_*S*_*j*__ = *k*_B_*T* ln(*NP*_*S*_*j*__/*D*_*S*_*j*__), with *N* the total number of molecules in the full system, which ensures that the segment equilibrium populations fulfill detailed balance without changing the effective rates. Here, *P*_*S*_*j*__ is the equilibrium population of segment *S*_*j*_ predicted by the TD-MCFRET method and *D*_*S*_*j*__ is the number of sites in segment *S*_*j*_, ensuring that *ΔE*_*S*_*j*__ = 0 when the rates are obtained in the high-temperature limit.

Next, we focus on the dynamics during *t*_3_ described by the window function. The system is now assumed to start in thermal equilibrium on segment *S*_*j*_ and the window function for segment *S*_*j*_ is
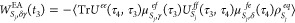
11Here, ρ_*S*_*j*__^eq^ is the equilibrium density matrix on segment *S*_*j*_ calculated as , where *H*_*S*_*j*__(*t*) is the time-dependent Hamiltonian at time *t* and ⟨ ··· ⟩ denotes the ensemble average over the full trajectory. The minus sign is taken from the overall sign of the EA signal. The window function is the same for the nonrephasing and rephasing diagrams. The full signal is given by products of the doorway, transfer, and window functions, summed over all combinations of segments.

In a similar way, we can obtain the doorway-window functions for each Feynman diagram. The doorway function in the nonrephasing part is identical for the GB, SE, and EA contributions. The same holds for the rephasing part, where
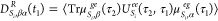
12which is the complex conjugate of the nonrephasing doorway function.

Furthermore, each window function is the same for the rephasing and nonrephasing contribution and only depends on if the signal is GB, SE, or EA. We will now consider the remaining window functions, again using the nonrephasing signals as examples.

The nonrephasing SE signal can be rewritten from eq 4 by separating the time-evolution operators so they only involve a single time interval, resulting in the expression
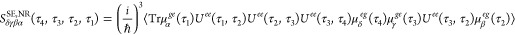
13From this, we identify the window function for the rephasing and nonrephasing SE signals
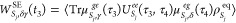
14

Finally, we move to the GB part, for which the nonrephasing signal has the form
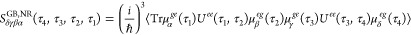
15The window function is then
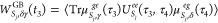
16Since the system resides in the ground state during waiting time *t*_2_, the window function for the GB signal does not depend on the equilibrium density matrix.

The total response function is the sum of the doorway-window signals. It can be expressed as follows:
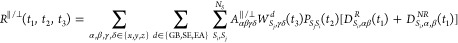
17Here, *N*_*S*_ is the number of segments, the symbol *d* represents the three diagram types (GB, SE, and EA) and α, β, γ, δ are representing Cartesian coordinates in the microscopic frame. *R* and *NR* represent rephasing and nonrephasing diagrams. (Note for the window functions, there is no difference between rephasing and nonrephasing, so the label is left out.) Finally, ∥and ⊥represent parallel and perpendicular polarization in the lab frame, respectively, and *A*_*αβγδ*_^∥/⊥^ are the weight factors connecting the microscopic frame with the lab frame; these values for an isotropic sample are given in ref (51). The parallel and perpendicular polarization spectra are obtained using the proper averaging over 21 nonzero polarization components (of the 81 possible Cartesian components) of the transition dipole vectors.^30,51,52^

## 3 Results

Here, we will employ the CG-2DES method described in the previous section to calculate the 2DES for different model systems, including one for which HEOM reference spectra and NISE spectra are already available.^53^ We further apply the CG-2DES method to the Light-harvesting 2 (LH2) system, which is one of the most studied photosynthetic complexes^36,54−56^ and is thus a suitable test system.

Simulations will be performed at room temperature (RT) and in the high-temperature (HT) limit. This distinction does not affect the bath parameters, which are always fixed and chosen in a range typical for RT systems. When considering the HT limit, we set the temperature in the density matrices to infinity, which results in an equal population of all sites within each segment and results in zero coherence between the molecules in each segment. We further apply the thermal correction of eq 9. The motivation for using the HT limit is that it allows for comparing the results directly with those obtained with the NISE method, which works in this limit, and it allows for assessing the importance of the thermalization effects by comparison with the finite-temperature result.

### 3.1 Single-Segment Dimer Test

First, we present the results of a 2DES study conducted on a dimer system with two sites (Figure 1 (b)) that have average energies corresponding to 11500 and 12000 cm^–1^ and a coupling of 100 cm^–1^ between the two. The parameters of the Hamiltonian have been taken from ref (53)., where they were carefully chosen to mimic a realistic light-harvesting system. In this subsection, we treat the entire dimer as one segment, i.e., we take into account the exact coupling between both molecules. To check the CG-2DES method, we have compared our results with those obtained using the HEOM method, which ensures the detailed balance of the quantum populations. To achieve this, we have used the overdamped Brownian oscillator model to generate the necessary bath fluctuations. A thorough explanation of the bath fluctuation scheme can be found in ref (36).

The simulation details are identical to the ref (53)., with the magnitude of the dynamic disorder σ = 198 cm^–1^ and a correlation time of Λ^–1^ = 220 fs. The classical coordinates are propagated using a time step *Δt* = 2 fs. The transition dipole moments for the two molecules were chosen to be parallel and their values were set to unity. All the calculations were run with the lengths of the trajectories of 10,000,000 time steps and with an average of over 100,000 realizations with starting times distributed equidistantly over the trajectory. The maximum coherence times for the doorway and window functions were set at 256 fs. The CG-2DES results were simulated at RT (300 K) and in the HT limit, which can be directly compared with the HEOM and NISE results, respectively.

**Figure 4:**
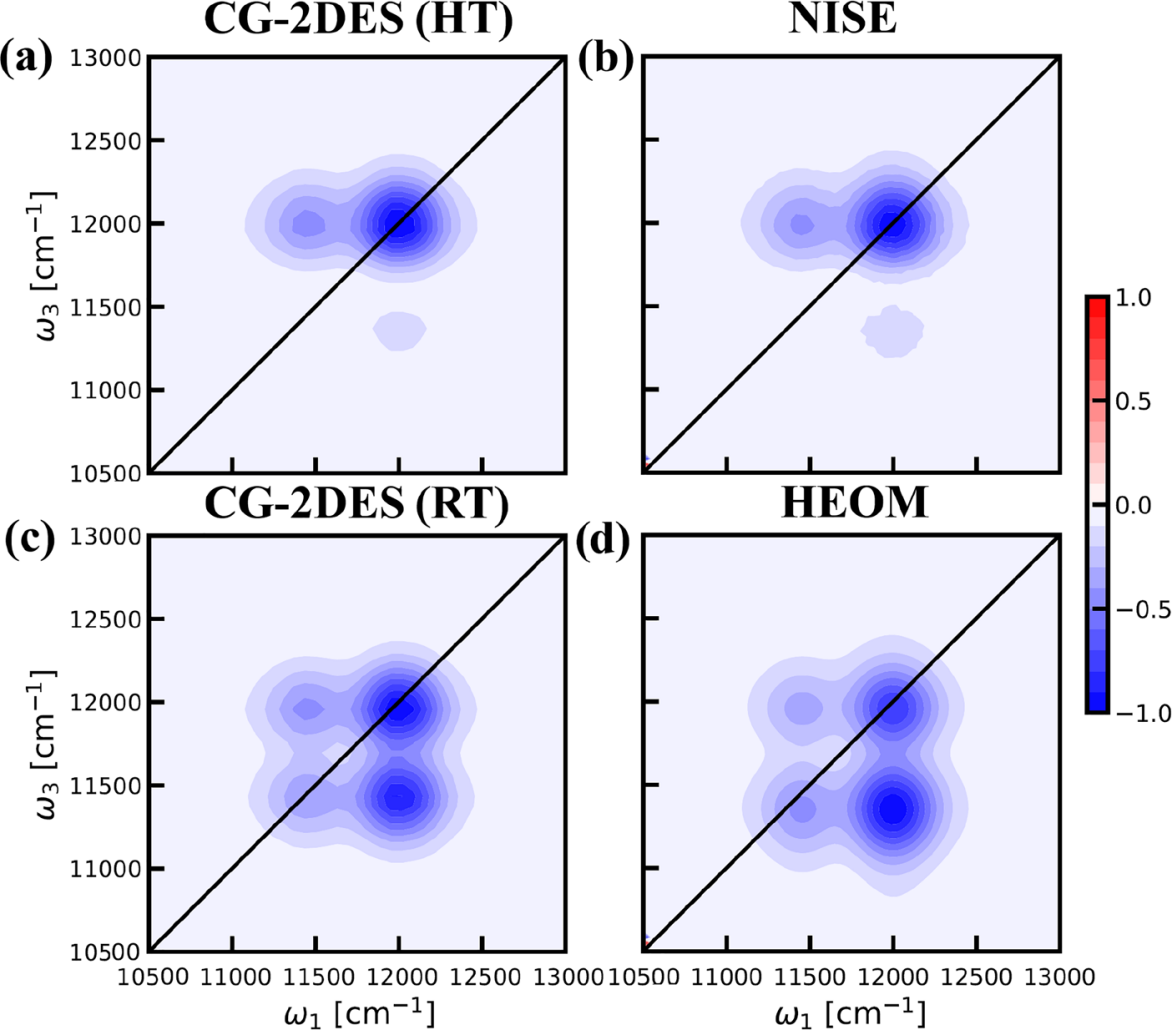
Calculated parallel polarization 2DES for the single-segment dimer system using the CG-2DES method compared with the NISE and HEOM results. (a) The 2DES was obtained in the HT limit with the CG-2DES method. (b) The 2DES simulated with the NISE approach, with the data taken from ref (53) at waiting time *t*_2_ = 15 ps. (c) The 2DES results calculated with the CG-2DES method at RT and 300 K. (d) The HEOM results obtained from ref (53) at 300 K at waiting time *t*_2_ = 15 ps. All data were normalized for each individual spectrum, and 20 equidistant contour lines were used in the range −1 to +1.

The resulting 2D spectra are shown as contour plots in Figure 4. The CG-2DES was calculated with a zero waiting time. We note that for the present method, the spectrum of an individual segment is independent of waiting time. Since the system only consists of one segment, and it corresponds to the spectrum expected after the equilibration of the segment. The spectra are, therefore, compared with NISE and HEOM spectra with a waiting time of 15 ps. The NISE spectra were found to have relaxed to the equilibrium state after 15 ps. As expected, the HT CG-2DES and the NISE spectra agree very well. This highlights the notable pros and cons of the CG-2DES approach. It efficiently calculates the spectrum of the equilibrium state. However, as formulated here, the approach does not provide any information about the dynamics toward equilibration within individual segments.

The relative intensity between the peaks in the simulated spectra may seem puzzling. As the transition dipoles are parallel and the coupling is positive, the high-frequency peak arises from the symmetric exciton state carrying the largest oscillator strength and the low-frequency one reflects the asymmetric state, carrying less oscillator strength. However, the actual intensities are further complicated by the interference of the GB and EA signals, which for the chosen parameters significantly suppress the peaks with ω_3_ around 11400 cm^–1^.

Moving on to the RT case, as seen in Figure 4, the CG-2DES method reproduces the benchmark HEOM results quite well. The CG-2DES approach neglects the Stokes shift, while the HEOM method includes it.^53^ This leads to the observed shift of the peaks to slightly lower detection frequencies in the HEOM results. Clear cross-peaks can be observed at RT, which is a manifestation of the population transfer between the two states. Further details of the analysis can be found in ref (53)., where this model was first used.

We, thus, find good agreement for the CG-2DES results with the reference results in both high- and finite-temperature cases as seen in Figure 4. This demonstrates that our developed CG-2DES method can be used to efficiently calculate the 2DES of equilibrated segments at different temperatures. While the current implementation of CG-2DES does not allow for the calculation of the equilibration dynamics within individual segments. One could include the intrasegment dynamics by combining the doorway-window function with NISE or HEOM calculations of the spectra for the cases where the segments before and after the waiting time are identical (*S*_*i*_ = *S*_*j*_) in eq 17.

### 3.2 Double-Segment Dimer Test

At longer waiting times, the CG-2DES method demonstrates good agreement with the NISE approach at higher temperatures in a one-segment model system, which is expected considering both methods employ the same propagation scheme. One notable advantage of the CG-2DES method is its ability to calculate the 2DES signal by summing all the signals for each segment, resulting in a significant reduction in computational resources. However, the exact extent of the speed improvement is difficult to quantify as it depends on the specific system and the segmentation applied. Additionally, the CG-2DES method incorporates temperature dependence without any HT limits. With this in mind, we conducted a test using a model system consisting of two segments to evaluate the method when incoherent transfer between segments is included.

The system’s Hamiltonian is represented by the same sites and parameters as in the single-segment dimer case studied above, except that the coupling between the sites is reduced to 10 cm^–1^ to ensure that the two-segments (here the two molecules in Figure 1 (c)) are weakly coupled and transfer between the sites is predominantly incoherent. The TD-MCFRET method^36^ was employed to generate the rate matrix for transfer between both molecules. In order to examine the thermalization effect on the 2D spectra, we again calculated the spectra at RT and HT. The corresponding rate matrices for the two temperatures are
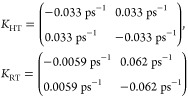
18The sum of the forward and backward rates are identical within numerical accuracy as this is imposed by the thermal correction of eq 9.

The *K*_HT_ represents the transfer rate matrix in the HT limit, where uphill and downhill energy transfer is equally probable, while for *K*_RT_ thermalization makes downhill energy transfer more likely. For a general number of segments, the diagonal elements of these matrices are the negative sum of the nondiagonal matrix elements in its column, with the upper off-diagonal elements denoting the transfer from the high-energy to the low-energy states and the lower ones representing the back transfer rate.

**Figure 5:**
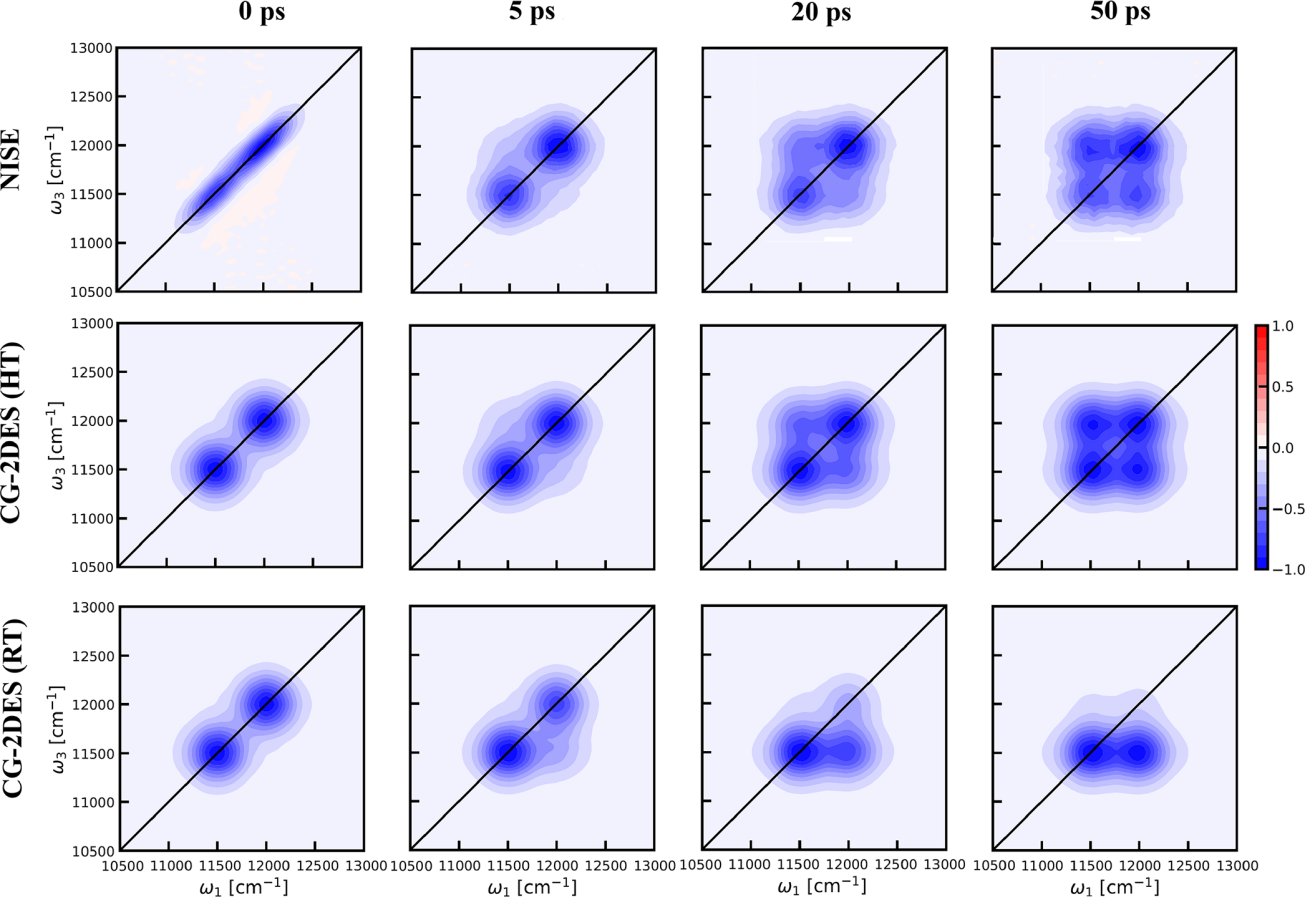
Calculated 2D spectra for a double-segment dimer system at waiting times *t*_2_ = 0 ps (first column), 5 ps (second column), 20 ps (third column), and 50 ps (fourth column). The first row displays results obtained using the conventional NISE method, which corresponds to the HT limit. Results for the CG-2DES approach in the HT limit are shown in the middle row. The bottom row shows the CG-2DES at RT and 300 K. Contours and normalization are as in Figure 4.

We show the 2DES in Figure 5 at four different waiting times. In all spectra, the intensity of the two cross-peaks increases with the waiting time, reflecting the energy transfer between the two segments. First, when focusing on comparing the NISE and CG-2DES, we observe some discrepancies between the results. At 0 ps waiting time, there is a noticeable difference in the line shape of the spectra. This can be attributed to the equilibration assumption within one segment before the transfer happens in the CG-2DES method. In contrast, for the NISE approach, the relaxation to the equilibrium state is included and at zero time, equilibrium has obviously not yet been reached, resulting in significant differences in the line shape of the diagonal peaks at 0 ps and to a limited extent for 5 ps, but not at later times. A sharp eye will identify slight intensity differences between the upper- and lower-frequency peaks for NISE caused by weak effect of the coupling resulting in intensity borrowing. This makes the intensity of the low energy state slightly higher than that of the high energy one.

The cross-peak dynamics, which with the purpose of the method and the approximations made, is the most interesting, is very well reproduced. At 20 ps waiting time, the cross-peak growth is significant. At 50 ps waiting time, equilibration is essentially complete. The only difference between the NISE and CG-2DES results from the intensity redistribution in the NISE spectrum discussed above.

A comparison of the two CG-2DES results readily reveals the effect of temperature on the 2DES. Initially, the spectra show no differences, which can be attributed to the equilibrium approximation within one segment and the fact that finite time is needed for transfer to take place. However, with increasing time delay, the RT case exhibits the growth of a lower cross-peak, while at the upper cross-peak position, the signal remains unchanged. This disparity arises from the transfer rate from the high-energy state to the low-energy state being more than ten times larger than the reverse transfer rate. A clear thermalization effect is thus observed, reflecting the incoherent exciton transfer described by the rate equation.

Summarizing the results so far, we found the CG-2DES method to work as expected and reproduce the results of other methods well at long waiting times where intersegment transfer takes place. We also saw that for short waiting times, the equilibration dynamics within segments are not reproduced. For short waiting times, it may be better to use other methods or possibly use more accurate calculations for the intrasegment contributions to the spectra. With respect to calculation efficiency, we found that to obtain the 2DES at a waiting time of 50 ps with the NISE and CG-2DES methods, using identical values for all simulation parameters as coherence times, number of realizations, etc., the NISE approach requires over three times more calculation time than the CG-2DES method for this particular situation of two segments.

### 3.3 Application to LH2

**Figure 6:**
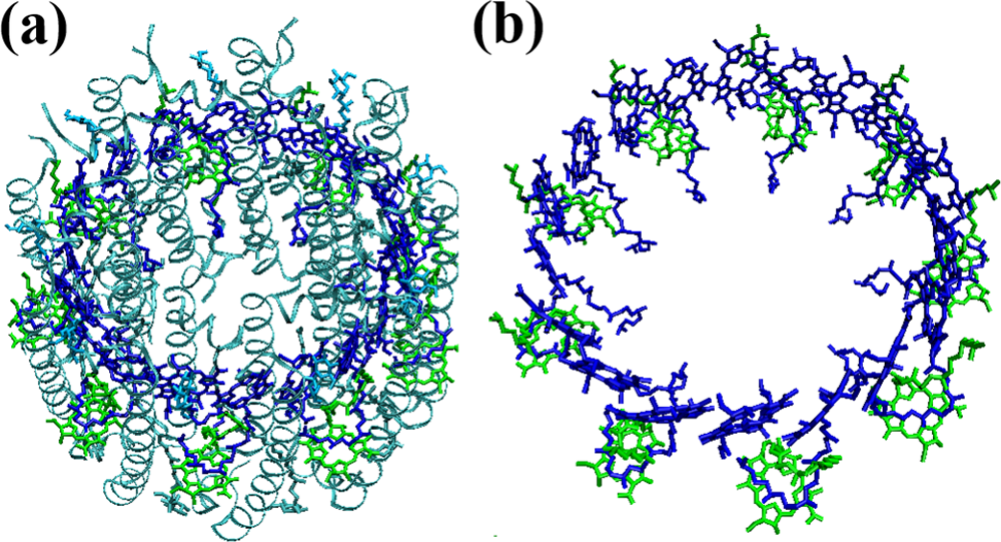
Nonameric structure of the peripheral light-harvesting complex (LH2) from *R*. *acidophilus* (PDB: 1KZU). (a) and (b) show the structure of LH2 with and without the protein scaffold, respectively. The protein scaffold is shown in turquoise. The B800 ring is depicted in green, and the B850 ring is represented in blue.

Having tested our method on different model systems, we will turn our attention to a realistic natural system, the LH2 system. This will allow us to further validate the CG-2DES method, as the LH2 system is particularly relevant to the light-harvesting process and is well-studied. The LH2 system is more complex than the above model systems, consisting of 27 chromophores, specifically Bacteriochlorophyll a (BChl a), arranged in two rings of 9 and 18 units. These chromophores are characterized by their excitation bands at 800 and 850 nm, respectively.^4,36,54,57,58^ For our analysis, we will focus on the *Rhodoblastus acidophilus* (formerly known as *Rhodopseudomonas acidophila*) as a representative bacterial system. The system is depicted in Figure 6. This bacterial system exhibits high symmetry and includes outer and inner B800 and B850 rings. In Figure 6, the protein scaffold, the B800 ring, and the B850 chromophores are highlighted. Figure 6 shows that the chromophores in the B850 ring are well connected, resulting in stronger excitation transfer interactions within the B850 ring compared to those within the B800 ring. In our model system, the largest nearest-neighbor coupling within the B850 ring is 243 cm^–1^, whereas it is only 30 cm^–1^ for the B800 ring.^36^ This larger coupling within the B850 ring leads to delocalized exciton states. Consequently, we consider all B850 chromophores as a single segment, and each individual B800 chromophore is treated as a separate segment. Thus, in total, we distinguish ten segments in the LH2 system.

In our study, we utilize the Frenkel exciton Hamiltonian eq 1 to describe the LH2 system. The bath dynamics is modeled using the overdamped Brownian oscillator model,^36^ with a correlation time of 150 fs for all the chromophores. The dynamic disorder magnitudes are set to σ = 256 and 169 cm^–1^ for the B850 and B800 chromophores, respectively. The excitonic couplings are determined using the TrEsp model,^59^ which was employed in our previous work.^36^ Additionally, we use the rescaling factor for the excitation transfer interactions caused by the dielectric constant with a value of 1/ε_*r*_ = 0.55, consistent with our previous work. Detailed information regarding the coupling and bath fluctuations can be found in ref (36).

In the HT limit, using the TD-MCFRET method, the rate of transfer from one B800 chromophore to the B850 segment was found to be 0.775 ps^–1^, with a backward rate of 0.0431 ps^–1^ (the two are related by a factor 18 in the HT limit). The transfer between neighboring B800 sites occurs at a rate of 1.83 ps^–1^, while the rate between B800 sites at larger separations are much smaller. At RT, the transfer rate from one B800 chromophore to the B850 segment increases to 1.48 ps^–1^, which is in reasonable agreement with other simulated results^60^ and experimental findings.^61,62^ The backward rate decreases significantly to 1.29 × 10^–3^ ps^–1^. The transfer between neighboring B800 sites remains the same at 1.83 ps^–1^, which is expected as these sites have the same energy on average.

We simulated 2DES using the NISE and CG-2DES methods at various temperatures and waiting times. These calculations were performed using a 3 ns long trajectory with starting points separated by 60 fs, and with a coherence time of 384 fs. The time step for each calculation was set to 3 fs. In the LH2 system, the B850 chromophores can be classified into two types depending on whether they are bound to the α-polypeptides or β-polypeptides. These α and β chromophores are known to have slightly different excitation energies.^63,64^ However, for simplicity in our model, we assume that all B850 chromophores have an identical average transition frequency of 11,955 cm^–1^, while all B800 chromophores (γ) have a transition frequency of 12,465 cm^–1^.^36^

**Figure 7:**
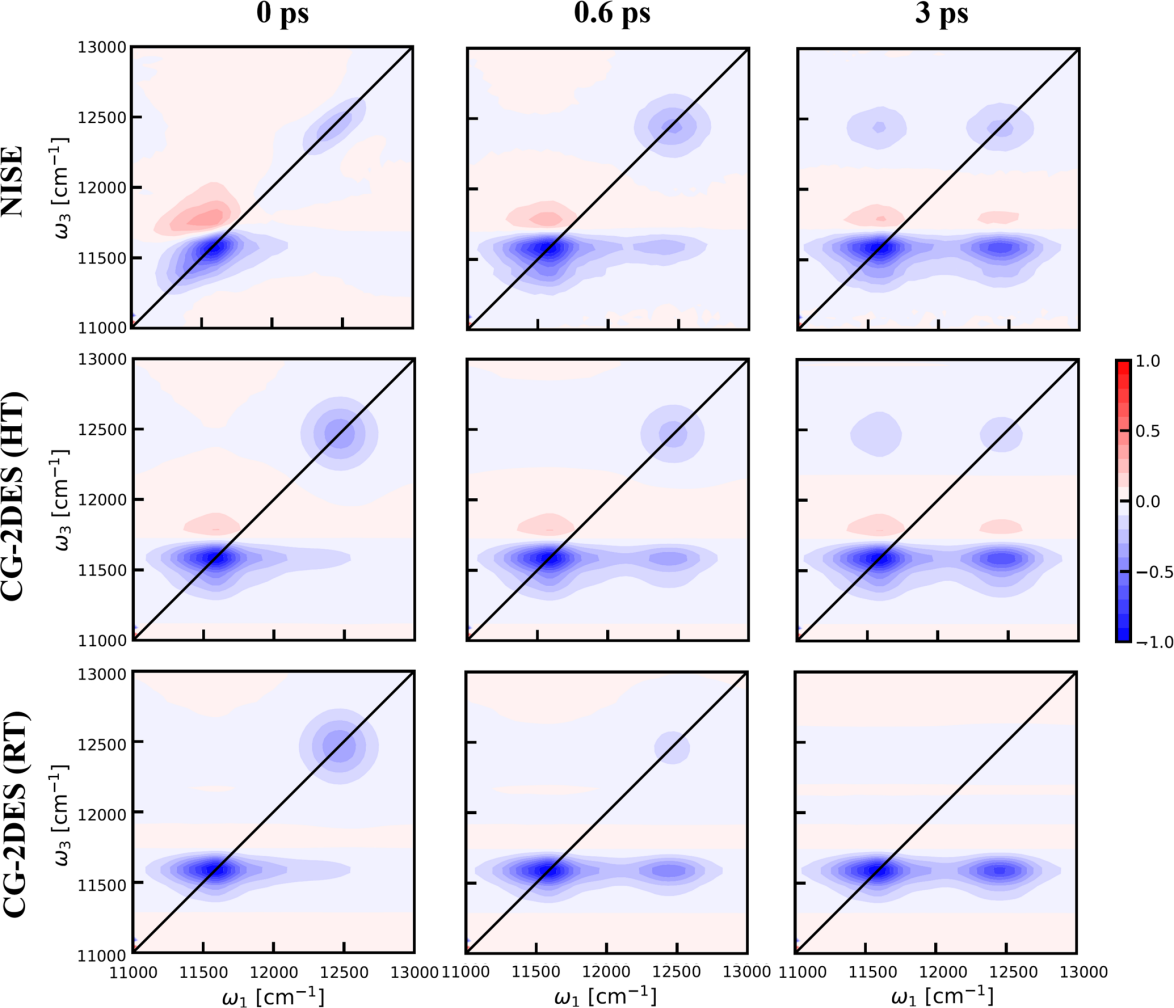
Parallel polarization 2DES calculated for the LH2 system at waiting times *t*_2_ = 0 (left column), 0.6 ps (middle column), and 3 ps (right column). The top row displays results obtained using the conventional NISE approach, whereas the middle and the bottom row are the results calculated with the CG-2DES method at HT and RT temperatures, respectively.

Figure 7 presents the 2DES results obtained using different methods and temperatures for the LH2 system. Let us first consider the HT case. Both the NISE and CG-2DES methods exhibit similar features in the spectrum. The two diagonal peaks correspond to the B800 ring (∼12,500 cm^–1^) and the B850 ring (∼11,600 cm^–1^). Notably, there is a distinct EA signal just above the diagonal peak of the B850 ring. This feature indicates the degree of delocalization of the excitons within the B850 ring.^65^ We also observe a difference in the line shape of the B800 peak between the NISE and CG-2DES methods. In the latter, the assumption of equilibrium within each segment results in a round peak shape, which remains the same over time. In contrast, the line shape of the B800 peak in the NISE results at short times is elongated along the diagonal and at longer waiting times, behaves similarly to that in the CG-2DES results as the waiting time increases. Furthermore, the CG-2DES results show a significant tail that extends to the cross-peak region. This is because we assume instant thermal equilibrium within the segments. In the LH2 system, there are higher energy levels than the optically active ones, which are already relaxed at time zero within our assumption, leading to tail elongation of the peak along the ω_1_-axis to the cross-peak area. In contrast to the CG-2DES method, the NISE line shape shows reasonable agreement with experimental results^66^ at short waiting times.

After a time delay of 0.6 ps, a clear cross-peak appears in both spectra in the lower right quadrant, reflecting the energy transfer from the B800 segment to the B850 ring. At a waiting time of 3 ps, the cross-peak becomes more pronounced while the intensity of the corresponding diagonal peak of the B800 rings decreases. These changes in the features are consistent with experimental results reported in the literature.^67−69^ Here, we also find that the computation time of the two methods differs significantly. To obtain 2DES at a waiting time of 3 ps, the NISE method requires 45 h of computation time on one CPU to complete the calculation, while the CG-2DES calculation only takes 17 h. This difference becomes more pronounced when we increase the waiting time. A noticeable difference can be seen when comparing the 2DES at RT with that at HT. It is important to note that, to compare the thermal effect, we only need to change the temperature in the thermal correction, eq 9, and in the equilibrium density matrix in these two cases, while keeping all parameters related to the bath fluctuations identical. The chosen Hamiltonian for the LH2 system changes the exciton delocalization. It affects the peak intensity, which is evident for the EA peak and results in a less prominent EA peak in the RT case. In our model system, the current TrEsp coupling model may overestimate the energy gap to a dark state^70^ known to lie below the optically active states. In RT equilibrium, the population of this dark state may thus be exaggerated. Therefore, the EA signal is less obvious when thermal relaxation is included. The NISE results for the LH2 system clearly show that with the increase in the waiting time, the EA signal also decreases significantly. It demonstrates that when the excitation relaxes from the optically active states to other states, it goes to dark states reducing the excited state absorption. However, other effects may contribute to the less prominent EA peak at RT. Improving the calculated contributions of EA processes may be achieved by accounting for the actual intrasegment dynamics, for instance by running the NISE calculation within each segment, and potentially by optimizing the Hamiltonian parameters using the calculated RT spectra. Nevertheless, from the comparison, it is clear that the RT spectrum demonstrates a stronger downhill transfer from the B800 band to the B850 band than the HT spectrum. This can be explained by the fact that the temperature-corrected downhill transfer rate at RT is twice as large as the HT rate, as seen above. Furthermore, the large disparity between the downhill transfer rate and the uphill transfer rate at RT leads to the absence of the upper diagonal peak at 3 ps, as also seen experimentally.

**Figure 8:**
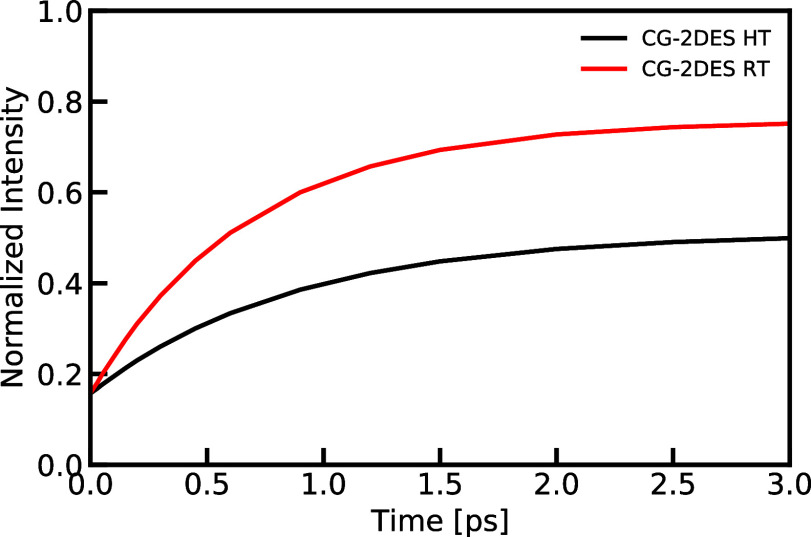
Comparison of the intensity growth of the below-diagonal cross peak as a function of waiting time, extracted from the 2DES for LH2 calculated using the CG-2DES method at different temperatures. The integration area to calculate the intensity of the process peak for ω_1_ was taken from 12,234 to 12,634 cm^–1^ and for ω_3_ from 11,400 to 11,650 cm^–1^. The data were normalized to the maximum diagonal peak intensity at *t*_2_ = 0 ps.

To better understand how the rate matrix affects the 2DES, we focus on the below-diagonal cross peak, which reflects the EET process. Figure 8 presents the growth of the cross peak intensity with increasing delay time, *t*_2_. The intensity changes can be fitted perfectly with a single-exponential function, which can be used to determine the intensity change rate. It is found to be 1.15 ps^–1^ and 1.48 ps^–1^ for the HT CG-2DES and the RT CG-2DES results, respectively. Interestingly, these rates align well with the corrected transfer rates (which is the eigenvalues obtained by diagonalizing the rate matrix) for the LH2 system, which are 1.16 ps^–1^ and 1.49 ps^–1^, respectively. This agreement between the cross-peak intensity increase rate and the transfer rate confirms the successful implementation of the transfer rate matrix in our CG-2DES simulation method. The agreement with the expected behavior is a good check that the implementation is working as expected. However, it should be noted that in the LH2 system, the transfer between the B800 chromophores involves a complex choreography of coherent and incoherent processes.^71^ Thus, when using different segmentation schemes, the transfer rate between both rings may vary. In this study, we divided the LH2 system into 10 segments (1 B850 segment and 9 B800 segments), resulting in a transfer rate of 0.775 ps^–1^ from a B800 chromophore to the B850 ring at HT. Conversely, when clustering the LH2 system into 2 segments (1 B850 segment and 1 B800 segment), the transfer rate from the B800 ring to the B850 ring is 0.705 ps^–1^.^36^ Notably, the eigenvalues of the rate matrix corresponding to transfer between the rings are found to be 1.15 ps^–1^ and 1.05 ps^–1^ (see ref (36).) for the 10-segment scheme and 2-segment protocol, respectively. This finding further supports the consistency between the cross-peak intensity change rate and the transfer rate.

**Figure 9:**
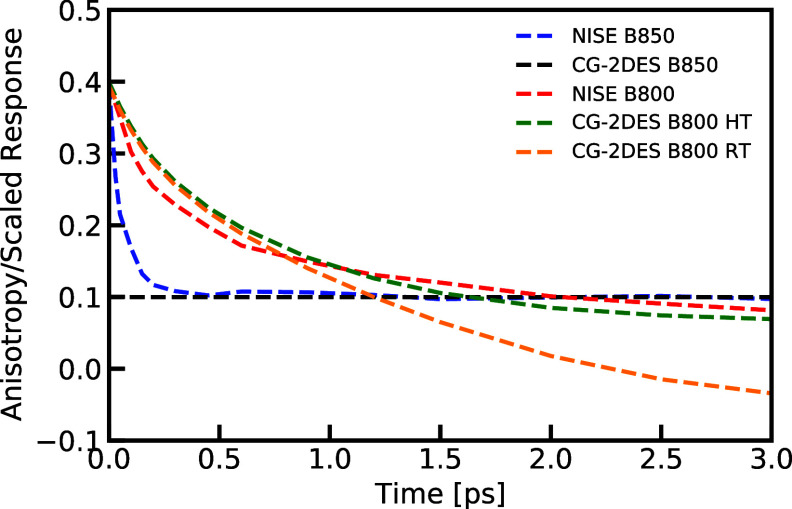
Comparison of the anisotropy decay as a function of waiting time extracted from the calculated 2DES for LH2 with the NISE and CG-2DES methods at different temperatures. The position of the B850 peak was taken to be ω_1_ = ω_3_ = 11,566 cm^–1^, and for the B800 peak was used ω_1_ = ω_3_ = 12,434 cm^–1^.

Furthermore, with the segmentation protocol, we can still look into the details of dynamic information within the LH2 system. We calculate the anisotropy from the simulation data near the diagonal B850 and B800 peaks using the relation , where *I*_∥_(*t*) and *I*_⊥_ are the intensities at waiting time *t* = *t*_2_ at the given spectral location for experiments with parallel and perpendicular laser pulse polarization, respectively.^51^Figure 9 illustrates the polarization anisotropy decay of the B850 ring and the B800 ring, simulated using the NISE and CG-2DES methods at various temperatures. The figure demonstrates that the anisotropy decay rate of the B850 ring is much faster than that of the B800 ring. The CG-2DES results for the B850 ring exhibit a single flat line, as in this method, we assume the population is in thermal equilibrium within a single segment at all times. By contrast, the NISE method does account for population dynamics between (or nonadiabatic mixing of) the eigenstates within the B850 ring, which causes the rapid decay of the anisotropy in this spectral region, as the transition dipole direction loses correlation in the plane. Moving on to the B800 ring, Figure 9 demonstrates that the HT NISE and CG-2DES results are very similar in terms of decay rate and the final equilibrium state. As for the RT case, the decay rate is also similar until about 1 ps waiting time, but it becomes negative thereafter, which has no physical meaning. This occurs because the B800 band completely relaxes to the low-energy B850 band after a waiting time of about 1 ps (see Figure 7). After this time, the background signal from the tail of the B850 band dominates the signal, resulting in a negative anisotropy value.

**Figure 10:**
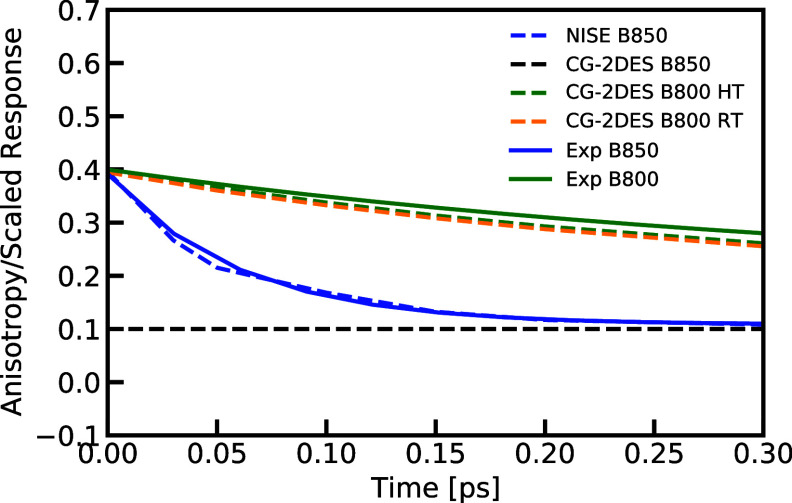
Polarization anisotropy as a function of waiting time obtained at the B850(NISE/HT CG-2DES) (blue/black dashes) and B800 (HT/RT) (green/orange dashes) diagonal positions of the two-dimensional electronic spectra compared with experimental data (solid lines). Experimental data were taken from ref (67)

Figure 10 compares the polarization anisotropy of the B850 and B800 bands with experimental results. Here we also added the benchmark line obtained from the B850 ring CG-2DES results as a reference. The solid lines represent the experimental decay for the B850 band (blue) and the B800 band (green). Experimental reports indicate decay times of 383 and 60 fs for the B800 and B850 bands, respectively.^67,72^ In our simulated results, the NISE method yields a decay time of 53 fs for the B850 ring, while the CG-2DES method yields a decay time of 302/306 (HT/*RT*) fs for the B800 ring (with fitted range from 0 to 0.3 ps). Both of these results agree well with the experimental findings, indicating that our developed CG-2DES method accurately captures the dynamic process.

## 4 Conclusion

In this paper, we introduced an approach, referred to as the CG-2DES method, for efficiently calculating third-order response functions in 2DES of large molecular systems. Our method separates the large system of molecules into a set of smaller segments, where the assumption is that the intermolecular excitation transfer interactions within segments are much larger than those between molecules of different segments. In fact, the assumption is that the latter are small enough to consider the energy transfer between segments as incoherent. In our method, we therefore use the previously developed TD-MCFRET method to construct an energy transfer rate matrix between the various segments. Solving the kinetics resulting from this rate matrix describes how excitations move between segments. Within each segment, we use the NISE approach to solve the collective excited state dynamics and we further assume that the excitations within segments relax much faster than the time scale of transfer between segments.

In particular, in the calculation of 2DES spectra, the coherence time *t*_1_ and *t*_3_ are dealt with by numerically propagating the exciton wave functions (NISE), accounting for interactions with a heat bath. The waiting time *t*_2_ is simulated using the kinetic rate equations described above, where it is assumed that the energy transfer between segments is slow enough that the excited state population within each segment reaches thermal equilibrium before transferring energy to another segment. In the CG-2DES method, the response signals are obtained as the sum of signals from each segment. This significantly reduces the computational resources required compared to calculating the response signal for the full system, which scales with N^3^, N being the number of excited states. Furthermore, with the current CG-2DES method, one can calculate the 2DES with additional waiting times within 1 min for the LH2 system, using the doorway and window functions already calculated and stored from a previous calculation with another waiting time. Moreover, our method incorporates the detailed balance condition, enabling simulations of 2DES at different temperatures.

To verify the performance and effectiveness of our method, we first demonstrated its application on a one-segment system at different temperatures and compared the results with the spectrum obtained using reference methods. The CG-2DES method showed excellent agreement with the NISE results at high temperatures and with HEOM results at low temperatures. We further applied the CG-2DES method to a two-segment system and compared it with the HT NISE results, revealing nearly identical spectral features. The low-temperature spectrum could be well understood using the transfer rate matrix.

Finally, we applied the CG-2DES method to the natural LH2 system, separating the system into ten segments. Our method demonstrates excellent performance and exhibits good agreement with theoretical NISE results. It also provides a clear visualization of the EET from the B800 band to the B850 band. Additionally, we calculated the anisotropy decay for the LH2 system, which accurately reproduced recent experimental results. The analysis of the anisotropy decay further confirmed that the CG-2DES method can effectively describe dynamic processes by replacing segment interactions with the transfer rate matrix.

Thus, we conclude that the CG-2DES method is an efficient method for accurately calculating the third-order optical response of large molecular systems if it is possible to separate the system into a set of smaller segments with relatively weak interactions between them.

The presented CG-2DES method thus offers a promising avenue for investigating the third-order response functions in 2DES of large molecular systems. Building upon the principles of system segmentation and energy transfer dynamics, this approach provides a framework for efficiently simulating complex molecular dynamics and elucidating the underlying mechanisms of exciton dynamics and energy transfer processes. Future improvements may include the explicit calculation of full single-segment spectra to include the frequency correlations observed at short times. Furthermore, one may find an effective way to include the Stokes shift missing in the stimulated and excited state signals, which may become significant at low temperatures. Moreover, in the future, we plan to include the intrasegment dynamics by combining the CG-2DES calculation with other methods for calculating the spectra of the individual segments.

## Data Availability

The data that support the findings of this study are available from the corresponding author upon reasonable request.
